# Acquiring “the Knowledge” of London's Layout Drives Structural Brain Changes

**DOI:** 10.1016/j.cub.2011.11.018

**Published:** 2011-12-20

**Authors:** Katherine Woollett, Eleanor A. Maguire

**Affiliations:** 1 Wellcome Trust Centre for Neuroimaging, Institute of Neurology, University College London, 12 Queen Square, London WC1N 3BG, UK

## Abstract

The last decade has seen a burgeoning of reports associating brain structure with specific skills and traits (e.g., [1–8]). Although these cross-sectional studies are informative, cause and effect are impossible to establish without longitudinal investigation of the same individuals before and after an intervention. Several longitudinal studies have been conducted (e.g., [9–18]); some involved children or young adults, potentially conflating brain development with learning, most were restricted to the motor domain, and all concerned relatively short timescales (weeks or months). Here, by contrast, we utilized a unique opportunity to study average-IQ adults operating in the real world as they learned, over four years, the complex layout of London's streets while training to become licensed taxi drivers. In those who qualified, acquisition of an internal spatial representation of London was associated with a selective increase in gray matter (GM) volume in their posterior hippocampi and concomitant changes to their memory profile. No structural brain changes were observed in trainees who failed to qualify or control participants. We conclude that specific, enduring, structural brain changes in adult humans can be induced by biologically relevant behaviors engaging higher cognitive functions such as spatial memory, with significance for the “nature versus nurture” debate.

## Highlights

► Trainee taxi drivers in London spend 3–4 years learning the city's layout ► We assessed the brain and memory of trainees before and after this long training ► Those who qualified experienced increased gray matter in posterior hippocampus ► Successful qualification was also associated with changes in memory profile

## Results and Discussion

**Figure 1:**
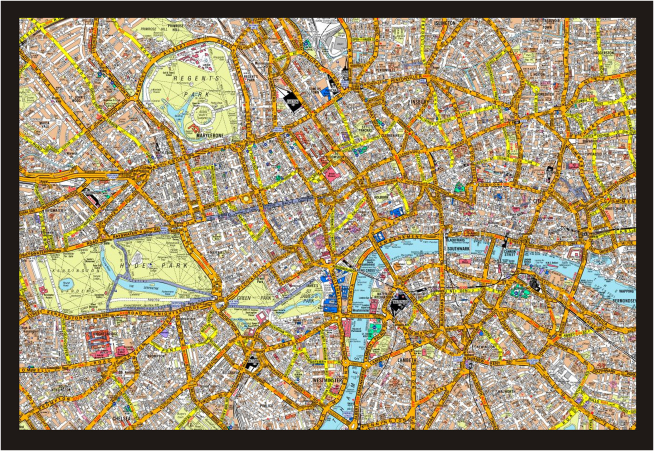
Central London Trainee taxi drivers must acquire “the Knowledge” of London's layout within a 6-mile radius of Charing Cross train station. This map shows just a part of the total area that must be learned. Map reproduced by permission of Geographers' A-Z Map Co. Ltd. © Crown copyright 2005. All rights reserved. License number 100017302.

In order to qualify as a licensed London taxi driver, a trainee must learn the complex and irregular layout of London's ∼25,000 streets (Figure 1) within a 6-mile radius of Charing Cross train station, along with the locations of thousands of places of interest. This spatial learning is known as acquiring “the Knowledge” and typically takes between 3 and 4 years, leading to a stringent set of examinations, called “appearances,” which must be passed in order to obtain an operating license from the Public Carriage Office (PCO, the official London taxi-licensing body). This comprehensive training and qualification procedure is unique among taxi drivers anywhere in the world. Previous cross-sectional studies of qualified London taxi drivers documented more gray matter (GM) volume in their posterior hippocampi and less in their anterior hippocampi relative to non-taxi-driving matched control participants [2–4]. Moreover, correlation of hippocampal GM volume with years of taxi driving suggested that structural differences may have been acquired through the experience of navigating, to accommodate the internal representation of London, and were not merely due to preexisting hippocampal GM volume patterns disposing individuals to being taxi drivers [2–4, 19, 20]. As well as displaying a specific pattern of hippocampal GM volume, qualified taxi drivers have been found to display better memory for London-based information, but surprisingly poorer learning and memory for certain types of new visual information (e.g., delayed recall of complex figures), compared with control participants, suggesting there might be a price to pay for the acquisition of their spatial knowledge, perhaps linked to their reduced anterior hippocampal volume (see [3, 4, 20] for more on this). Interestingly, the opposite pattern of hippocampal GM volume and memory profile has been described in retired taxi drivers, hinting that any changes acquired through learning might be reversed or “normalized” when the call on stored memory representations lessens [21].

London taxi drivers are therefore a useful model of memory, illuminating the role of the hippocampus and intrahippocampal functional differentiation and potentially informing about whether hippocampal structure and memory capacity are hardwired or amenable to change. Given current economic imperatives and increasing longevity, the need to keep retraining and learning throughout adulthood has never been more acute. Direct evidence for hippocampal plasticity in response to environmental stimulation could allow us to understand the boundaries within which human memory operates and the scope for improving or rehabilitating memory in educational and clinical contexts. Moreover, given the dearth of longitudinal magnetic resonance imaging (MRI) structural association studies focusing on higher cognitive functions in average adults engaged in truly naturalistic behaviors, taxi drivers could contribute new information to the wider debate about whether key aspects of cognition are fixed or malleable.

The above aspirations are predicated upon hippocampal plasticity in adult humans, evidence for which remains sparse [10, 18]. We therefore conducted a longitudinal study examining 79 male trainee London taxi drivers at the start of their training (T1, time 1) and then again 3–4 years later just after qualification (T2, time 2), as well as 31 male control participants, with two aims. First, given that the PCO suggests that typically 50%–60% of trainees fail to qualify, we anticipated having three groups of participants: trainees who qualified (Q), trainees who failed to qualify (F), and the controls (C). With this design, we could retrospectively examine whether MRI and/or neuropsychological findings at T1 could predict who would eventually qualify 3–4 years later at T2. Second, we sought to ascertain whether the pattern of hippocampal GM volume and memory profile observed in previous cross-sectional taxi driver studies would be induced and observable within the same participants as a result of acquiring “the Knowledge.”

Of the 79 trainees, 39 went on to qualify as licensed London taxi drivers, while 20 did not qualify (ceased training or failed their appearances) but agreed to return for testing at T2. Of the other 20 trainees who did not wish to return at T2, two had qualified, and the remaining 18 decided to stop training or made no appearances. Thus in our sample, 51.9% of trainees qualified, in line with PCO figures. All 31 control participants returned for testing at T2.

### Time 1

We first focused on the data acquired at T1 and examined the status before training occurred of those trainees who subsequently qualified or not, as well as the control participants. There were no statistically significant differences between the three groups on a range of background measures such as age, handedness, education, and IQ [F(8,166) = 1.81; p = 0.07; see Table 1]. We next looked at their performance at T1 on a battery of tests assessing working and long-term memory, recognition and recall, and visual and verbal material (see Supplemental Experimental Procedures and Table S1A available online). There were no statistically significant differences between the three groups for any of the memory measures [F(18,158) = 1.33; p = 0.18]. Finally, we examined the structural MRI brain scans of participants acquired at T1, before training. An automated whole-brain analysis method, voxel-based morphometry (VBM; [22, 23]) implemented in SPM8, was used to compare GM volume between the groups. No significant differences were found, even with a liberal statistical threshold (p < 0.005 uncorrected for multiple comparisons).

Our data therefore showed that at T1, before acquisition of a detailed internal spatial representation of London's layout, there was no general intellectual, mnemonic, or structural brain difference that was associated with subsequent success at qualification. In particular, hippocampal GM volume, both anteriorly and posteriorly, was indistinguishable between those who would qualify, those who would not, and the controls. This means that the groups started out on equal terms and that any changes that subsequently emerged would be due to acquiring “the Knowledge.”

### Time 2

In the first instance, we compared the two trainee groups on a range of training-related variables (see Table 1). There was no significant difference between those who qualified and those who did not in terms of the total time they had spent in training [t(57) = 0.67; p = 0.5]. There was a difference, however, in the number of hours per week the groups spent training, with the trainees who qualified spending twice as many hours per week training compared to the group who failed to qualify [t(57) = 6.62; p = 0.001]. Unsurprisingly, the two groups differed also in the number of appearances that they made, with the failed candidates making hardly any [t(57) = 13.1; p = 0.001]. Finally, there was no significant difference in the time elapsed between testing at T1 and testing at T2 for the Q, F, and C groups [F(2, 86) = 1.27; p = 0.28].

We next examined performance at T2 on parallel versions of the memory tests that had first been employed at T1 (see Table S1B). Whereas at T1 there were no differences between the groups, at T2 this was no longer the case: significant differences between the three groups now emerged [F(18,158) = 2.35; p = 0.004], driven by two main effects. A significant difference was found between the groups on the London landmarks proximity judgments test [F(2,87) = 8.09 p = 0.001], with trainees who qualified being significantly better at judging the spatial relations between London landmarks than the control participants (p = 0.003), as were nonqualified trainees (p = 0.003). The groups also differed on the delayed recall of the Taylor complex figure [F(2,87) = 4.38; p = 0.015], with qualified trainees being significantly worse at recalling the complex figure after 30 min delay than the control participants (p = 0.01). By contrast, the performance of the nonqualified trainees was not significantly different from that of control participants (p = 0.14).

The memory profile displayed by the now qualified trainees mirrors exactly the pattern displayed in several previous cross-sectional studies of licensed London taxi drivers [3, 4, 20] (and that which normalized in the retired taxi drivers [21]). In those studies also, the taxi drivers displayed more knowledge of the spatial relationships between landmarks in London, unsurprisingly, given their increased exposure to the city compared to control participants. By contrast, this enhanced spatial representation of the city was accompanied by poorer performance on a complex figure test, a visuospatial task designed to assess the free recall of visual material after 30 min. Our findings therefore not only replicate those of previous cross-sectional studies but extend them by showing the change in memory profile within the same participants. That the only major difference between T1 and T2 was acquiring “the Knowledge” strongly suggests that this is what induced the memory change.

**Figure 2:**
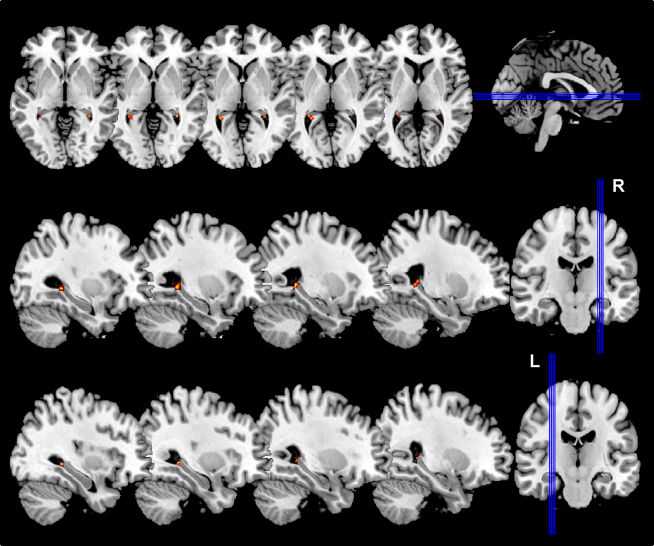
Gray Matter Volume Changes between T1 and T2 in Qualified Trainees Gray matter (GM) volume increased in the most posterior part of the hippocampus bilaterally between T1 and T2 as a result of acquiring a detailed representation of London's layout. This change was only apparent in the trainees who qualified. The upper row shows axial views, and the middle and lower rows show sagittal views through the right (R) and left (L) sides of the brain, respectively, that encompass the GM changes (shown in orange and yellow) in the peak voxels detailed in the text.

**Figure 3:**
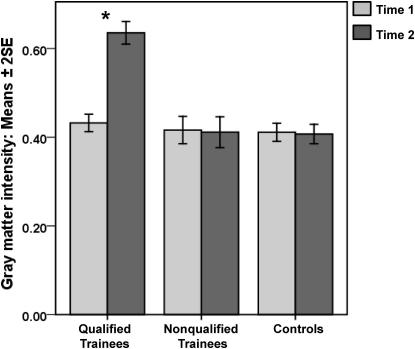
Plot of Gray Matter Intensities across Groups and Time GM intensity values were extracted from the four peak hippocampal voxels identified by the HDW procedure (see text for details). Here we show a plot from one of those peaks (the others resulted in very similar plots) at 20, −37, 10 in the right posterior hippocampus. Only the qualified trainees experienced a significant increase in posterior hippocampal gray matter between T1 and T2; this change was evident in every qualified trainee. Data are presented as means ± two standard errors of the mean. ^∗^p < 0.05.

We then turned to the structural MRI brain scans acquired at T2 and compared them to the scans acquired in the same individuals at T1 in order to assess whether acquiring “the Knowledge” had any impact on GM volume. This was accomplished by implementing high-dimensional warping (HDW) in SPM8. HDW safeguards against nonspecific subtle differences that may arise between the first and second scans within subjects in a longitudinal study (see Supplemental Experimental Procedures and also [24] for full details). The time between scans, total GM volume, and participant age were modeled as confounding variables. Given our a priori interest in the hippocampus, the significance level was set at p < 0.001 corrected for the volume of the hippocampus; otherwise the significance level was set at p < 0.05 corrected for multiple comparisons across the whole brain. We first looked at the scans of the qualified trainees and found that GM volume had increased in the posterior hippocampi bilaterally (30, −42, 1, z = 5.44; 20, −37, 10, z = 5.64; 24, −39, 7, z = 4.02; −29, −42, 3, z = 5.91) at T2 relative to T1 (see Figure 2 and Figure 3). The opposite comparison (T1 > T2) did not show any significant differences, nor were there any significant differences in GM volume anywhere else in the brain for either contrast. Similar analyses were performed for the trainees who failed to qualify. No significant differences in GM volume were found anywhere in the brain, including the hippocampi, for T2 > T1 or T1 > T2. When the data from the two time points of the control participants were compared, again no significant differences in GM volume emerged.

As with the memory data, the structural MRI brain data replicate and extend the cross-sectional taxi driver studies [2–4] by showing that the increased posterior hippocampal GM volume previously observed most likely occurred as a result of acquiring the detailed spatial representation of London's layout. Importantly, the posterior hippocampal change in GM volume cannot be attributed to the qualified trainee taxi drivers' training procedures, methods, or general attempts to learn, as the trainees who failed to qualify were exposed to the same milieu. Although the posterior hippocampal increase accords with previous findings, we did not observe a decrease in anterior hippocampal GM volume. This is interesting, because it may provide an insight into the time frame of the hippocampal structural changes. It could be that they occur serially, with the increase in posterior hippocampus happening first and within 3–4 years. We speculate that the anterior hippocampal GM volume might then decrease subsequently and in response to the posterior increase. In fact, the poor performance of the qualified trainees on the delayed recall of the complex figure at T2 may be an indication that changes are already afoot in the anterior hippocampus but are not yet detectable with MRI.

That acquiring “the Knowledge,” which encompasses spatial learning and memory, can drive changes in posterior hippocampus illustrates the close relationship between this region and spatial navigation [25–27] and suggests that the hippocampus acts as a storage site for the spatial information acquired during “the Knowledge,” or as a processing hub for detailed navigational information. Our results underline the existence of functional differentiation in the hippocampus, with anterior and posterior regions diverging in their response to spatial memory [28, 29]. The hippocampal plasticity we have observed in vivo in adult humans parallels the effects reported in nonhumans where intraindividual hippocampal volume changes occur in response to demands placed on spatial memory [30, 31]. Having documented this hippocampal change, the question is what mechanism underpins this process.

Using standard structural MRI scanning in humans, it is not possible to address this question directly, but based on work in nonhumans, there are several candidates. Studies in rodents have demonstrated that when learning requires cognitive effort and where learning actually takes place (i.e., where material is remembered after a delay), there is an effect on the rate of hippocampal neurogenesis [32]. Moreover, the animals that learn best have more new neurons after training than those who do not learn, or do not learn efficiently [33, 34]. If neurogenesis is what underpins the hippocampal volume change in qualified taxi drivers, it may be related to recruitment of new neurons following neurogenesis [35, 36] that are pressed into the service of spatial memory. The development of greater communication between neurons in the form of increased synaptogenesis [37, 38] might also be involved, and proliferation in dendritic arborization, augmenting connectivity between neurons, could in turn increase memory capacity and also lead to volumetric changes [39]. Glial cells, which continue to be produced, albeit at a slow pace, throughout adulthood [40], could also be implicated and have been shown to increase in volume with the addition of synapses following learning [41]. In the future, new approaches to human brain scanning in vivo may eventually be able to provide more direct insight into the key mechanisms supporting human hippocampal plasticity [42].

To conclude, we have shown that there is a capacity for memory improvement and concomitant structural changes to occur in the human brain well into adulthood. That there are many thousands of licensed London taxi drivers shows that acquisition of “the Knowledge,” and presumably the brain changes that arise from it, is not uncommon, offering encouragement for lifelong learning, and possibly a role in neurorehabilitation in the clinical context. However, this needs to be balanced by our finding that memory improvement in one domain may come at the expense of memory performance elsewhere. One final point to consider concerns the PCO figures and our data showing that only half of trainees who undertake “the Knowledge” actually qualify. The reasons we were given for ceasing training included the time commitment being too great, financial imperatives, and family obligations. Very few trainees reported ceasing because they found the spatial memory demands to be too great, although it is possible, or even likely, that the reasons given may have masked such difficulties in some individuals. It could be that there are inherited factors that feed into individual differences in spatial memory and navigation ability. One source of influence may come from genes. Genetic association studies have demonstrated effects of specific gene polymorphisms on the volume of the hippocampus [43, 44] and memory performance [45, 46]. Although our data show that environmental stimulation can drive structural brain changes, it may be that this hippocampal plasticity expresses itself only in certain individuals. The trainees that qualified may have had a genetic predisposition toward plasticity that the nonqualified individuals lacked. Thus, the perennial question of “nature versus nurture” is still open for future investigations that incorporate genetic and other influences on individual differences, as well as cognitive and structural brain dimensions.

## Experimental Procedures

All participants gave informed written consent to participation in accordance with the local research ethics committee. Full details of the participants, cognitive tests, MRI scans, and data analysis procedures are provided in the Supplemental Experimental Procedures.

## Figures and Tables

**Table 1:** Background Characteristics of the Participants

Measure	Qualified (n = 39)	Failed to Qualify (n = 20)	Controls (n = 31)
Age (years)	37.97 ± 7.96	40.50 ± 5.27	35 ± 8.99
Age left school (years)	16.66 ± 1.32	16.75 ± 1.40	16.77 ± 1.30
Estimated verbal IQ	97.72 ± 6.29	98.66 ± 3.49	100.79 ± 3.79
Matrix reasoning (scaled score)	11.89 ± 2.06	12.20 ± 1.98	11.83 ± 2.39
Handedness (laterality index)	87.97 ± 24.09	88.10 ± 21.50	71.74 ± 38.69
Total training time (months)	38.84 ± 7.02	35.80 ± 10.32	–
Training time per week (hours)^a^	34.56 ± 12.40	16.70 ± 8.21	–
Number of appearances^a^	15.64 ± 3.66	2.60 ± 3.45	–
Time between T1 and T2 testing (months)	35.28 ± 8.19	36.15 ± 8.53	32.8 ± 7.37

Measurements are given in means ± SD.

a Significantly more for trainees who qualified compared to trainees who did not qualify.

## Supplemental Information

Document S1. One Table and Supplemental Experimental Procedures
