# Supplementary material for: Acquiring “the Knowledge” of London's Layout Drives Structural Brain Changes
Source: Curr Biol. 2011 Dec 20;21(24-2):2109–14. doi: 10.1016/j.cub.2011.11.018 (PMC3268356; doi:10.1016/j.cub.2011.11.018)
Supplement: Document S1. One Table and Supplemental Experimental Procedures [file mmc1.pdf]

Current Biology, Volume *21*

Supplemental Information

Acquiring "the Knowledge" of London's Layout Drives Structural Brain Changes

Katherine Woollett and Eleanor A. Maguire

Table S1. Scores on the Memory Measures

Measures (means, SD)	Qualified (n=39)	Failed to Qualify (n=20)	Controls (n=31)
A. Time 1			
**Digit span (scaled score)**	12.15 (3.26)	12.80 (3.31)	12.19 (2.52)
**Recognition memory faces (scaled score)**	11.71 (3.75)	12.10 (2.88)	10.29 (3.29)
**Recognition memory words (scaled score)**	12.51 (1.53)	13.20 (1.36)	12.38 (1.62)
**Story recall, immediate (/56)**	40.74 (6.38)	40.95 (8.35)	43.41 (8.56)
**Story recall, delayed (/56)**	39.51 (6.35)	38.60 (8.38)	40.90 (8.29)
**Rey complex figure, copy (/36)**	35.51 (1.07)	35.80 (0.61)	35.87 (0.49)
**Rey complex figure, delayed recall (/36)**	22.39 (5.99)	23.35 (4.20)	25.01 (5.98)
**London landmarks proximity judgements (/10)**	7.69 (1.34)	8 (1.12)	7.38 (1.43)
B. Time 2			
**Digit span (scaled score)**	11.12 (2.83)	11.10 (2.61)	12.29 (2.84)
**Recognition memory faces (scaled score)**	13.28 (3.16)	13.00 (2.82)	12.38 (2.59)
**Recognition memory words (scaled score)**	12.43 (1.95)	13.70 (0.92)	12.41 (1.85)
**Story recall, immediate (/60)**	31.35 (10.09)	33.20 (10.45)	34.64 (11.72)
**Story recall, delayed (/60)**	29.53 (11.29)	32.65 (9.92)	33.58 (11.33)
**Taylor complex figure, copy (/36)**	35.92 (0.48)	35.75 (0.91)	35.93 (0.24)
**Taylor complex figure, delayed recall (/36)** [C]	21.93 (5.90)	22.60 (4.29)	25.77 (5.81)
**London landmarks proximity judgements (/10)** [Q, F]	8.51 (0.94)	8.75 (1.25)	7.32 (1.97)

[C] = controls significantly better than the qualified trainees.
[Q, F] = both groups of trainees significantly better than controls.

Supplemental Experimental Procedures

Participants

The participants, all healthy males, had normal or corrected-to-normal vision and gave informed written consent to participation in accordance with the local research ethics committee. Trainee taxi drivers were recruited in cooperation with the Public Carriage Office (PCO) and a number of 'Knowledge schools', the taxi driver training schools in London. All were verified as officially registered 'Knowledge' candidates with the PCO. None had ever worked as mini-cab drivers. Similarly, none of the control participants had ever worked as a licensed London taxi driver, or been involved in taxi-driver training, nor had they ever worked as mini-cab drivers. None had attended university, in order to match education level of taxi trainees, and did not engage in any educational learning courses during the course of the study. They were recruited from the Centre's volunteer database, from public service departments in local government offices, local banks, and via public libraries. None of the participants, trainees or controls, had taken part in previous studies at the Centre.

Procedure

Testing at each time point (T1/T2) took place in a session of approximately 2.5 hours. The order of the cognitive assessment and MRI brain scan within a session was random across participants. Between T1 and T2 contact was maintained with participants twice-yearly in order to monitor the training and number of appearances of the trainees, and any changes in circumstance for both trainees and controls.

Cognitive Tests

All participants had excellent spoken and written English. The vast majority of participants were native English speakers and as such, an estimate of verbal IQ was obtained using the Wechsler Test of Adult Reading [WTAR; 1]. In order to assess visual information processing and abstract reasoning skills, and also to gain a measure of intellectual functioning in participants who were not native English speakers, the Matrix Reasoning sub-test of the Wechsler Abbreviated Scale of Intelligence [WASI; 2] was used. Handedness was assessed with the Edinburgh Handedness Inventory [3].

A range of other memory tasks were also employed (see Table S1). Note that different versions of the tests were used at T1 and T2. The tests were: Digit Span from the Wechsler Adult Intelligence Scale III [WAIS III; 4] which tests working memory and attention. Warrington Recognition Memory for Words [5] assesses recognition memory for words. Warrington Recognition Memory for Faces [5] assesses recognition memory for faces. Adult Memory and Information Processing Battery (AMIPB) Story Recall subtest [6] is very similar to the logical memory subtest of the Wechsler Memory Scale (but with UK normative data), and is designed to assess the free recall of verbal material at immediate and 30 minutes delayed recall. Rey-Osterrieth Complex Figure [7-8] (Taylor Complex Figure at T2 [9-10]) - this test is designed to assess perceptual organization, when the figure is copied, and the free recall of visual material when retrieved from memory 30 minutes later. A London landmark proximity judgements test was used to examine participants' knowledge of the spatial relationships between London landmarks. Stimuli were 10 colour photographs each depicting a London landmark. On each trial, subjects had to judge which of two other London landmarks was closer (as the crow flies) to the target London landmark. There were 10 trials. The test was not formally timed, however participants on average took 5-8 seconds per photograph.

Basic group comparisons were made using two-tailed t-tests or ANOVAs. For the main analyses, data were screened for outliers, homogeneity of variance, and to ascertain if the data were normally distributed. Multivariate analysis of variance (MANOVA - Hotelling's trace multivariate test) was employed using the general linear model with the significance threshold set at $p<0.05$. Where a

MANOVA indicated a significant effect, the between-subjects tests were employed to ascertain the source of the significance with a threshold of $p<0.05$.

MRI Scan

Whole brain structural MRI scans were acquired on a 1.5 T Sonata whole body scanner (Siemens Medical Systems, Erlangen, Germany), with a whole-body coil for RF transmission and an 8-element phased-array head coil for signal reception, using a Modified Driven Equilibrium Fourier Transform (MDEFT) sequence [11]. Each participant had two scans, one at Time 1 (start of training) and another at Time 2, using the same scanning sequence. The parameters were optimised as described in the literature [12-14]: for each volunteer, 176 sagittal partitions were acquired with an image matrix of 256 x 240 (Read x Phase). Two-fold oversampling was performed in the read direction (head/foot direction) to prevent aliasing. The isotropic spatial resolution was 1 mm. Relevant imaging parameters were TR/TE/TI = 14.59 ms / 3.4 ms / 650 ms, BW = 96 Hz/Px, $\alpha = 20°$. To increase the signal-to-noise ratio, an asymmetric position of the inversion pulse within the magnetisation preparation (duration TI) was chosen, and the delay between the initial saturation and the inversion amounted to 40% of TI [14]. A fat saturation pulse was used to achieve fat suppression (see [14] for details). In addition, special RF excitation pulses were used to compensate for B1 inhomogeneities of the transmit coil [13]. Images were reconstructed by performing a standard 3D Fourier transform, followed by modulus calculation. No data filtering was applied either in k space or in the image domain. The total duration of a scan was 12 minutes.

MR images were analysed using VBM implemented in SPM8, using DARTEL for the individual time point analyses, and employing a smoothing kernel of 8mm full width at half maximum. Analyses focussed on grey matter. The effects of global grey matter volume and subject age were excluded by modelling them as confounding variables. Given our *a priori* interest in the hippocampus, the significance level was set at $p<0.001$ corrected for the volume of the hippocampus, otherwise the significance level was set at $p<0.05$ corrected for multiple comparisons across the whole brain. For the comparison of T1 and T2 scans, high dimensional warping (HDW) was implemented SPM8. HDW safeguards against non-specific subtle differences that may arise between the first and second scans within subjects (for full details see [15]). In brief, a rigid registration of early to late scans is performed. This is followed by high dimensional registration which results in a detailed deformation field containing mapping from each point in the late image to its corresponding point in the early image for each participant. This is achieved by matching the images while concurrently maximising the smoothness of the deformations. The smoothness is incorporated into the registration using Bayesian statistics to optimise finding the maximum a posteriori (MAP) estimate of the deformation field of the parameters [16]. Grey matter changes are estimated individually before spatial normalization thus providing greater sensitivity. The arising deformation images were segmented and normalised.

Scanner stability was monitored throughout the study, and quality assurance carefully documented. The scanner was very stable, with no significant changes in the geometry of the images, the signal-to-noise ratio, signal stability or signal drift.

Supplemental References

1. Wechsler, D. (2001). Wechsler Test of Adult Reading. The Psychological Corporation: San Antonio, TX.
2. Wechsler, D. (1999). Wechsler Abbreviated Scale of Intelligence. The Psychological Corporation: San Antonio, TX.
3. Oldfield, R.C. (1971). The assessment and analysis of handedness: The Edinburgh inventory. Neuropsychologia *9*, 97-113.
4. Wechsler, D. (1997). Wechsler Adult Intelligence Scale III. The Psychological Corporation. San Antonio, TX.
5. Warrington, E.K. (1984). Manual for Recognition Memory Test. Windsor, England: NFER-Nelson.
6. Coughlan, A.K., and Hollows, S.E. (1985). The Adult Memory and Information Processing Battery Test Manual. Leeds, UK: Psychology Department, University of Leeds.
7. Rey, A. (1941). L'examen psychologique dans les cas d'encephalopathie traumatique. Archives de Psychologie *28*, 286-340.
8. Osterrieth, P.A. (1944). Le test de copie d'une figure complexe. Archives de Psychologie *30*, 206–356.
9. Taylor, L.B. (1979). Psychological assessment of neurosurgical patients. In T. Rasmussen and R. Marino (Eds.) Functional Neurosurgery. New York: Raven Press.
10. Hubley, A.M., and Tremblay, D. (2002). Comparability of total score performance on the Rey-Osterrieth Complex Figure and a modified Taylor Complex Figure. Journal of Clinical and Experimental Neuropsychology *24*, 370-382.
11. Ugurbil, K. et al. (1993). Imaging at high magnetic fields: initial experiences at 4 T. Magnetic Resonance Quarterly *9*, 259-277.
12. Deichmann, R., Good, C.D., and Turner, R. (2002). RF inhomogeneity compensation in structural brain imaging. Magn. Reson. Med. *47,* 398-402.
13. Deichmann, R., Schwarzbauer, C., and Turner, R. (2004). Optimisation of the 3D MDEFT sequence for anatomical brain imaging: technical implications at 1.5 and 3 T. Neuroimage *21*, 757-767.
14. Howarth, C., Hutton, C., Deichmann, R. (2006). Improvement of the image quality of T1-weighted anatomical brain scans. Neuroimage *29*, 930-937.
15. Kipps, C.M., Duggins, A.J., Mahant, N., Gomes, L., Ashburner, J., McCusker, E.A. (2005). Progression of structural neuropathology in preclinical Huntington's disease: a tensor based morphometry study. J. Neurol. Neurosurg. Psych. *76*, 650-655
16. Ashburner, J., Andersson, J.L., and Friston, K.J. (1999). High-dimensional image registration using symmetric priors. Neuroimage *9*, 619-628.
